# A deep learning approach for brain tumour classification and detection in MRI images using YOLOv7

**DOI:** 10.3389/fonc.2025.1508326

**Published:** 2025-09-17

**Authors:** Ramya Nimmagadda, P. Kalpana Devi

**Affiliations:** Electronics and Communication Engineering (ECE), Vel Tech Rangarajan Dr. Sagunthala R&D Institute of Science and Technology, Chennai, India

**Keywords:** YOLOv7, brain tumour, MRI, classification, object detection, deep learning

## Abstract

The medical imaging field has grown tremendously due to the latest digital imaging and artificial intelligence (AI) advancements. These advancements have improved tumour classification accuracy, time, cost efficiency, etc. Radiologists utilize an MRI scan due to its exceptional capacity to identify even the most minor alterations in brain activity. This research uses YOLOv7, a Deep Learning (DL) model, to classify and detect brain tumours and to conduct a detailed analysis of the frequently used structures for tumour identification. The study uses a brain MRI dataset from Roboflow with 2870 labelled pictures divided into four types of tumours. Our brain tumour dataset has four distinct classes: pituitary, gliomas, meningiomas, and no tumours. This preprocessed sample was used to assess the performance of deep learning models on identifying and classifying brain tumours. Throughout the preprocessing stage, aspect ratio normalization and resizing algorithms are applied to improve tumour localization for bounding box-based detection. YOLOv7 performs admirably, with a recall score of 0.813 and a box detection accuracy of 0.837. Remarkably, the mAP value for the 0.5 IoU threshold is 0.879. During box identification within the extended IoU spectrum of 0.5 for a to 0.95, the mAP value was 0.442.

## 1 Introduction

As per the classification system of brain tumors by the World Health Organization (WHO), there are over 120 types of brain tumors that exist on the basis of their origin, location, size, and the characteristics of the tissues that constitute the tumor. These brain tumors can be divided into two types: malignant (cancerous) and benign (non-cancerous). Some tumors can be aggressive in nature, and others can be inactive. However, if there is a sufficient increase in size, the tumor will compress the adjacent nerves and blood vessels and thus impair the normal brain functions and can also kill the brain cells (1). Abnormally strong deformities of the tissues cause brain tumors. Tumors stem from cell clusters that arise within the brain as a result of excessive cell multiplication. These clusters can affect normal brain functions and can cause the destruction of healthy brain cells. Several of the body’s processes, such as integrating, organizing, evaluating, and decision making, are regulated by the central nervous system (CNS), which consists of the spinal cord and the brain. The astonishing detail of an individual’s brain arises from its complex structure (2). There is a range of illnesses that can affect the CNS, including brain tumors, migraines, infections, and strokes, which pose substantial challenges in the area of screening, evaluation, and effective treatment development (3). The abnormal development of brain cells creates brain tumors, which pose substantial challenges for radiologists and neuropathologists in early diagnosis. The primary difficulty confronting radiologists and neuropathologists is the early detection of brain tumors resulting from the rapid development of certain neurons. One of the most common types of imaging used for the diagnosis of brain tumors is magnetic resonance imaging (MRI), which often proves to be inaccurate and unreliable, particularly for these sensitive tumors. An uncharacteristic proliferation of nerve tissue forming a mass is usually a constitutive trait of malignancies in the porto systems. Brain tumors have close to 130 types, and some are highly unusual, but most are fairly common. Tumors are classified into benign and malignant. They can arise from neurons, oligodendrocytes, and other supportive cells that encase the adjacent nerve cells. The most important form of malignant brain tumor is the so-called metastatic or secondary brain tumor. Benign tumors do not spread to other parts of the body, but when they do, they can cause significant health problems, thus turning malignant (4).

Gliomas, meningiomas, pituitary tumors, and no tumors represent almost all of the primary tumors diagnosed. Most cases of meningiomas start in the blood vessel cells on the exterior of the central nervous system, which arise from the outermost layers enveloping the central nervous system. Even so, the brain tumor type that kills people the fastest is glioma, which starts in the tissues that protect neuronal activity. Gliomas account for approximately one-third of all brain tumor cases. Benign pituitary tumors grow inside the pituitary gland. The prognosis and available treatments for brain tumors depend heavily on a reliable evaluation. Traditional biopsy methods are uncomfortable, time-consuming, and prone to inaccurate sampling. There are a number of issues with histopathological tumor grading (biopsy), such as intra-tumor heterogeneity and variations in the expert’s opinions. These qualities make the tumor diagnosis procedures hard and limited.

Identifying the tumor accurately and in a timely fashion is critical in planning any form of therapy and achieving the required clinical outcome. For brain tumors in particular, a good deal of work may be conducted by radiologists at the interpretation stage. Now, radiologists have to identify and diagnose using images, so in a way, they are limited to their subjective assessments.

Due to the intersection of the complexity of the images with variable skill levels of clinicians, making an accurate diagnosis through individual sensory judgment is exceptionally rare for brain tumors. For neurology, MRI is a preferred method since it allows for the detailed evaluation of the brain and skull. It produces sagittal, coronal, and axial images for a comprehensive review. MRI not only is capable of producing highly detailed and contrast-rich images but also does not expose the patient to radiation risks, which makes it ideal. This is why in the diagnosis of a range of different types of brain tumors, MRI is highly recommended as a screening method.

The low accuracy of MRI in tumor detection has led to a need for automated methods that combine image processing and machine learning (ML). Soft tumors and extremely challenging tumors require different therapeutic approaches. The proposed You Only Look Once (YOLO) model is in keeping with artificial intelligence being increasingly integrated into healthcare. It provides potential advantages in efficiency and accuracy. The approach, findings, and outcomes of the present research are covered completely in the following parts, which also highlight its significance to the fields of medicine and computing. Current procedures take much time to complete and may not be entirely accurate due to human variance. In order to close this gap and meet the demand for accurate and timely brain tumor evaluation, this study suggests a sophisticated brain tumor categorization method based on the YOLO approach.

This study aims to maximize tumor identification efficiency using artificial intelligence frameworks, specifically the YOLO model. Among the objectives are modifying YOLO for the detection of malignancies in evaluating changes and optimizing parameters for training time and sensitivity. Enhancing the accuracy of tumor identification and categorization is the aim of this research, which will help the computer and medical industries. In this introduction, the background is established, the importance of automated tumor identification is emphasized, and YOLO is presented as a possible remedy.

Due to the challenges inherent in the traditional methods of diagnosing brain tumors and the automated techniques published in the literature, this study was framed around the following critical questions: How flexible is the YOLOv7 architecture for the accurate detection and classification of various types of brain tumors in MRI scans? How does its efficiency measure in comparison to other ML and deep learning (DL) models in terms of accuracy? Moreover, what modifications can be made to the architecture or other parameters to enhance the effectiveness of YOLOv7 for medical imaging tasks such as brain tumor segmentation? All answers are provided in Section 6.

In Section 2, the discussion highlights the previous research works conducted on brain tumors while incorporating various machine learning techniques. Section 3 provides a detailed overview of the study including the methodology, proposed model, and architecture best suited for our research. The deep learning frameworks and efficiency indicators employed for the research are also assessed. Section 4 discusses the results of our analysis on the performance of the deep learning algorithms. An extensive and detailed analysis is presented in Section 5.

## 2 Related works

The advancements of artificial intelligence (AI) technologies, especially in deep learning, show promising capabilities regarding the automation of the identification and classification of brain tumors in medical imaging, such as MRI scans. The literature offers a variety of approaches from models specialized in segmentation and classification-centric architecture to more modern ones that attempt to merge both tasks. Also, some studies have explored explainable hybrid optimization models. This article intends to narrate the overview blocked range under the thematic subheaders outlining the literature.

### 2.1 Tumor segmentation

The early diagnosis and timely intervention of a brain tumor case are heavily reliant on the precise segmentation of tumors from MRI scans. The manual process of segmentation comes with overly optimistic promises, as it is both labor-intensive and heterogeneous in nature (inter-rater reliability). For these reasons, automated deep learning models are gaining popularity.

SegNet is a fully convolutional network that has been used in the automated segmentation of necrosis, edema, and enhanced tumor regions alongside other multi-modal MRI sequences (T1, T1ce ( T1-weighted contrast-enhanced MRI), T2, and FLAIR (Fluid-Attenuated Inversion Recovery, a specific type of magnetic resonance imaging (MRI) sequence), like a tumor’s substructures. Researchers reported impressive F-measure outcomes of 0.85, 0.81, and 0.79 for whole, core, and enhancing tumors, respectively (5).

In the same way, a Mask R-CNN with DenseNet-41 backbone was created to perform the segmentation and categorization of tumors simultaneously, thus achieving an improved tumor boundary precision through transfer learning (6).

The more recent segmentation work conducted by Kamnitsas et al. (2023) introduced a dual-pathway 3D convolutional neural network (CNN) ensemble for high-resolution multi-view segmentation, which was integrated with CRF (Conditional Random Fields) post-processing for spatial coherence, thus achieving excellent results on the BraTS dataset.

### 2.2 Tumor classification

Some studies have focused exclusively on classifying the tumors based on the specific features given. A 23-layer CNN was trained on a multi-class MRI dataset with 3,064 and 152 images for “case-based” and “control” groups, respectively, thus demonstrating the power of CNNs on large datasets and their weaknesses on smaller ones (7). To enhance the performance on smaller datasets, the model applied transfer learning using VGG16.

Another study integrated min–max normalization and dropout layers into EfficientNet for the multi-class classification of pituitary tumor, meningioma, glioma, and no tumor to enhance performance and mitigate overfitting (8).

YOLO-based architectures have also been adopted for classification due to their efficiency in real-time object detection. Studies using YOLOv5 and YOLOv7 have reported over 99% accuracy in classifying meningioma, glioma, and pituitary tumors using MRI datasets from King Khaled University Hospital (9, 10).

A recent benchmark study conducted by Karthik et al. (11) (as mentioned in **Table 1**) compared YOLOv5, YOLOv6, and YOLOv7 and reported 87.9% classification accuracy with YOLOv7 which outperformed earlier versions and even the classical methods like Faster R-CNN with VGG16.

**Table 1:** Comparison of various studies and models and their results.

Study	Model	Accuracy	Interpretability	Limitation
Rao et al. (12)	CNN + RNN (LSTM)	~96%	Moderate	Slow, not real-time
Karthik et al. (11)	YOLOv7	87.90%	None	No attention module
Proposed	YOLOv7 + CBAM + SPPF+	**99.50%**	**High (Grad-CAM)**	Best performance, real-time

The Bold Values in **Table 1** highlights : the Accuracy percentage (99.50%) and Interpretability levels (High) of our proposed model which is the highest among all the compared models and has given the best performance of all the compared models and that too in real time.

### 2.3 Segmentation and classification

Integrated frameworks that perform segmentation and classification in tandem have been proposed by multiple researchers. Tumors were segmented and classified using the FAHS-SVM (Fully Automatic Heterogeneous Segmentation using Support vector machine) method, which applied deep learning as well as structural and morphological information (13).

In a different approach, YOLOv5 was applied in a two-step pipeline for real-time brain tumor segmentation and classification, achieving an 85.95% detection rate (14).

The study by Bhanothu et al. (2020) used Faster R-CNN and VGG16 for detection and classification in parallel and obtained classification accuracy of 89.45% for meningioma, 75.18% for glioma, and 68.18% for pituitary tumors (15).

More recent work by Isensee et al. (2024) utilized nnU-Net with dynamic adaptation for both segmentation and classification across multiple datasets and offered improved generalizability across tumor types and institutions.

### 2.4 Explainability and interpretability

“Black-box” issues have been associated with deep learning models despite the advanced accuracy associated with them, which has brought much criticism. Very few research studies have focused on this issue.

One remarkable attempt utilized Grad-CAM-based visualization approaches to highlight attention focus areas in MRI scans during tumor classification by the models. This method enhances clinical trust and provides some level of interpretability of AI decisions.

In another study, attention-based CNNs were used to increase transparency by assigning importance weights to different MRI regions, thereby enabling clinical validation and aiding radiologists in identifying diagnostic features.

### 2.5 Hybrid and optimization approaches

To improve the model’s accuracy and efficiency, hybrid models coupled with refinement methods have been introduced. In one of the studies, the parameters of a CNN were optimized with an Adaptive Dynamic Sine-Cosine Grey Wolf Optimizer alongside Inception-ResNetV2 for improved feature extraction and convergence rate (16).

Another work proposed a hybrid brain tumor classification (HBTC) model that combined handcrafted features from MGLCM (Modified Gray Level Co-occurrence Matrix) with deep learning for improved classification performance using a tree-based classifier for final voxel-level labeling (17, 18).

Transfer learning approaches were also explored extensively using pre-trained weights from the COCO dataset to train YOLOv4-Tiny models on the RSNA-MICCAI BraTS 2021 dataset (19), thus resulting in faster convergence and better generalization.

### 2.6 Limitations in related studies

Despite the attempts made to apply DL models such as YOLO and CNNs or even hybrid models for the detection and classification of brain tumors, there still remain significant issues and gaps in these models, as detailed below.

#### 2.6.1 Data imbalance and limited dataset diversity

A number of research works use openly accessible datasets like BraTS, which have class imbalance and lack of diversity, and the same imaging protocols. In addition, if a model is trained on non-homogeneous clinical data, it can become overfitted to real-life data. Most studies do not tackle the problem of the depiction of less common varieties of tumors or the different population-based heterogeneity in patient’s tumor demographics.

#### 2.6.2 Poor performance for small or asymmetric tumors

As highlighted in several investigations, models like YOLOv3–v7 and even newer versions such as YOLOv8 tend to struggle with detecting very small or irregularly shaped tumors, particularly when tumors blend with surrounding tissues. These detection issues are critical, especially in early diagnosis, where small tumors carry high clinical importance.

#### 2.6.3 Reliance on high-quality annotated data

Radiology machine learning models require a large amount of data that have been annotated with great precision. This poses issues as radiologists annotating the data by hand, which is quite tedious and time-consuming, and automated tagging tools are often prone to inaccuracies. In models that are trained on sparse annotations (for example, Mask R-CNN with DenseNet), poor ground truth annotations will invariably weaken the performance of the model.

#### 2.6.4 Lack of standardized evaluation metrics and validation protocols

Different studies have applied varying reporting standards and evaluation criteria (using metrics such as accuracy, precision, and F1 score), which hampers the fair benchmarking of models. Moreover, in many instances, there is no cross-validation or external validation, which reduces the trust in the claimed robustness of the model.

#### 2.6.5 High computational demands and latency concerns

Healthcare facilities may not possess the advanced GPUs (Graphics Processing Unit) required for computationally intensive training of models like Inception-ResNetV2, EfficientNet, and deeper CNN architectures (for example, a 23-layer CNN), making it challenging to use these AI systems in practice. While systems like YOLOv5 and YOLOv7 are slightly optimized, the ability to perform tasks in real time is still severely impacted in constrained resource environments.

#### 2.6.6 Limited real-time clinical integration

Some models, such as YOLOv5 in Dipu et al., show potential in real-time settings, but most works do not consider evaluating these models in operational clinical settings within workflows. This remains an open area not just from the ease of automation perspective but also from a regulatory, privacy, and workflow standpoint.

#### 2.6.7 Generalization across imaging modalities and institutions

Many models are developed and validated with very limited sets of imaging modalities, such as T1-weighted or T1ce. However, different brands of MRI scanners, acquisition protocols, and even how the patient is positioned can create domain shifts, which may impact the model’s in-line performance.

#### 2.6.8 Lack of explainability and trust at clinical level

Even though accuracy metrics are emphasized, there are very few studies that explain problems or provide interpretability tools such as Grad-CAM or saliency maps, which would enable clinicians to appreciate the reasoning behind the model.

#### 2.6.9 Inconsistent performance on multi-class classification

In multi-class tumor types, the differentiation of glioma, meningioma, and pituitary tumors is usually more complex than binary classification. Accuracy for different types of tumors is not at the same level as shown in cases like the study of Bhanothu et al. There is significant divergence, which suggests unreliable performance or bias toward more common classes.

#### 2.6.10 Neglect of segmentation refinement and post-processing

Certain works make use of the segmentation models like SegNet or FAHS-SVM but do not incorporate any boundary refinement procedures that would reduce segmentation errors or improve boundary delineation. The precision of tumor boundary detection is critical for planning the treatment, and clinical utility is diminished if boundary uncertainty is not resolved. When choosing a deep learning model for brain tumor segmentation using MRI images, many factors should be considered, such as its performance, architectural design, precision, and ease of modification according to the specific requirements of the project.

### 2.7 Rationale for choosing YOLOv7

Some of the commonly used models for segmentation in medical imaging are U-Net, DeepLabV3+, and Attention U-Net. These models target pixel-level identification and perform well on medical imaging segmentation tasks. However, these attention models can be slower than YOLOv7 in achieving real-time results due to the high computations required for segmentation processing.

Transformer-based models such as Swin Transformer or ViT are becoming more common for segmentation tasks. However, they are slower to train and more demanding in terms of memory utilization, thus making them less than ideal in limited-resource settings.

Therefore, while the newer versions of YOLO or other architectures may offer some incremental improvements, YOLOv7 was selected for our specific MRI scan dataset for its ability to offer a good balance between real-time performance, high detection accuracy with fast inference speed, and ease of its adaptation to our specific needs. YOLOv7 provided better accuracy in detecting and classifying brain tumors, especially small or irregularly shaped ones, as compared to other models like YOLOv5, YOLOv8, U-Net, and Faster R-CNN.

## 3 Proposed approach

### 3.1 Overall architecture of brain tumor detection

**Figure 1:**
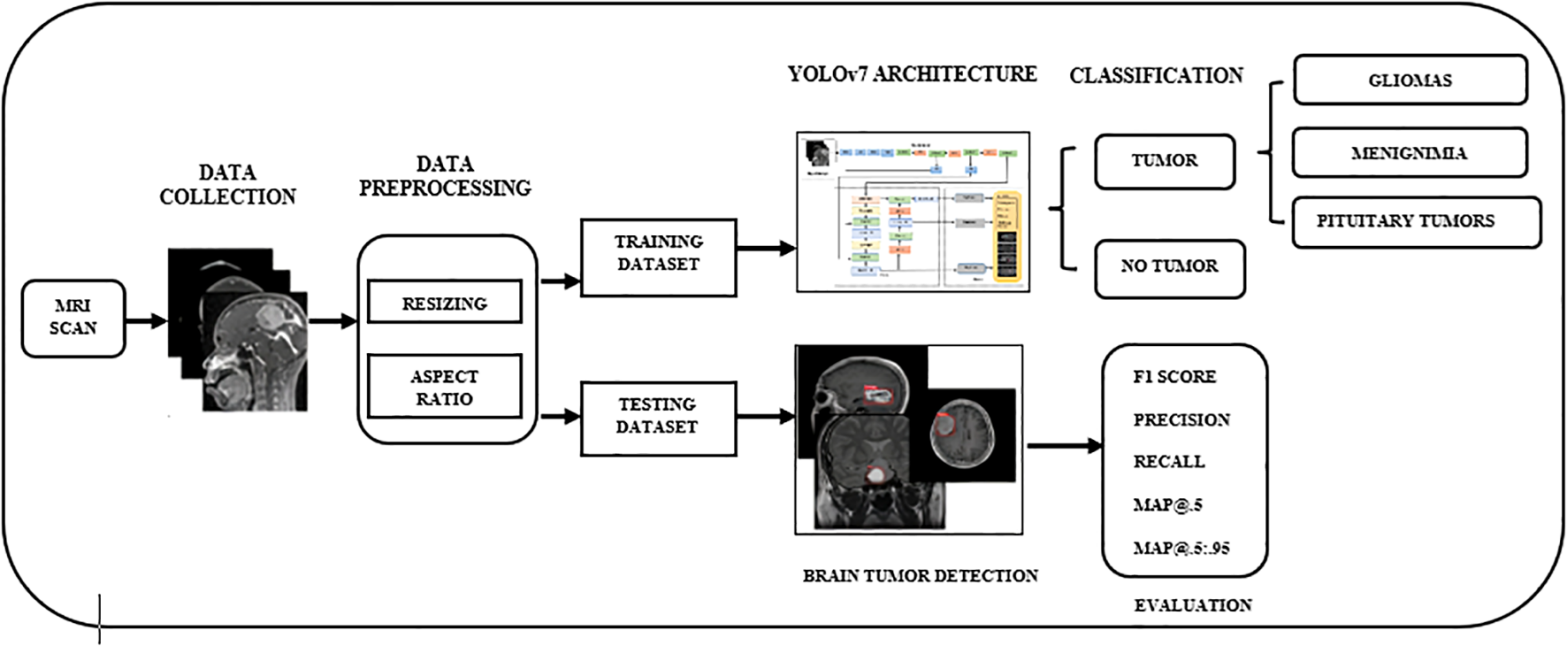
The overall workflow of YOLO-based brain tumor segmentation and classification. YOLO, You Only Look Once.

The evaluation of visualizations of brain tumors is challenging due to the size, shape, and positioning of the disorders. Scholars have come up with different ways to identify the anomalies in data, and each has its own advantages and disadvantages. Various machines are capable of producing images of brain tumors with differing levels of contrast, sharpness, number of slices, and spatial resolution. Here, we discuss the scientific specifications and architectural design of the algorithmic framework for the efficient and accurate image-based detection of brain tumors. With the recommended approach, we aim to differentiate malignant tumors in MRI scans with precision. **Figure 1** showcases the preprocessing, training, and evaluation steps conducted on images containing tumors. Considering YOLOv7’s proven performance for detecting brain tumors, this study selected it as the primary framework.

### 3.2 Dataset collection

To validate the accuracy of our findings, we used an MRI dataset containing 2,870 brain images sourced from Roboflow (20). MRI scans provide the highest accuracy possible in identifying brain tumors, which is why they have been included in this set. Our dataset, comprising brain tumors, had four subsets, as follows: no tumor (327 images), meningioma (823 images), pituitary tumor (834 images), and glioma (886 images). For this particular analysis, we selected approximately 70% of the entire dataset, equaling 2,009 MRI scans for training, 10% (287 MRI images) for testing, and 20% (574 MRI images) for validation.

#### 3.2.1 Dataset structure

This dataset is organized into four classes, which represent both the presence and absence of tumors:

Class Number of images Description.

No tumor 327 MRI scans of healthy individuals with no visible brain abnormalities.

Meningioma 823 Images containing meningioma, which are typically benign tumors of the meninges.

Pituitary tumor 834 Scans containing tumors in the pituitary gland region.

Glioma 886 MRI scans with gliomas, aggressive tumors originating in glial cells.

To facilitate the model training process and meet the requirements of the deep learning frameworks, all images were cropped to a standard size of 512 × 512 pixels. In addition, the resizing process simplifies the computational burden while maintaining the relevant features of the brain scans.

#### 3.2.2 Dataset splitting

To create a strong and effective model, the dataset was partitioned into three subsets:

Training set: 70% of the data (2,009 images).

Validation set: 20% of the data (574 images).

Test set: 10% of the data (287 images).

This partition guarantees that the model has adequate information to be trained on while also being validated and tested on new data to measure its ability to generalize.

#### 3.2.3 Data imbalance

A notable issue in the dataset was the class imbalance, as seen in the number of images.

The “no tumor” class contained only 327 images, which were significantly fewer than those of the other tumor classes (meningioma, pituitary tumor, and glioma), each having over 800 images.

The class with the highest number of images, glioma (886), had more than 2.7 times the images in the no tumor class.

This imbalance poses several challenges.

Model bias: The model may become biased toward tumor classes, particularly glioma, due to its over-representation, while underperforming on under-represented classes like no tumor.

Reduced sensitivity and specificity: The classifier may show lower accuracy for detecting healthy cases, resulting in a higher false-positive rate.

Skewed performance metrics: High overall accuracy may be misleading if the model fails to correctly predict the minority class.

#### 3.2.4 Addressing the data imbalance

To address the class imbalance, the following techniques were considered.

Data augmentation: Geometric and photometric transformations (such as rotations, flips, zoom, and brightness adjustments) were applied to increase the effective size of the minority class.

Class weighting: Class weights were set during the model training phase and were set inversely to the frequency of the class to increase the penalty for the wrong classification of minority classes.

Sampling strategies: Oversampling of minority classes and/or undersampling of majority classes was conducted to attain a suitable class distribution during training.

These strategies aim to enhance the model’s ability to generalize across all classes and reduce bias toward over-represented tumor types.

### 3.3 Data preprocessing

As a means of preparing the dataset to be suitable for classification tasks, a number of preprocessing steps were performed to standardize the images of brain tumors. Below is a list of the preparatory processes that were performed: RGB images underwent grayscale conversion in order to create a single monochrome image. This can simplify the image data, which in turn will reduce computational requirements. All images were scaled to a uniform resolution of 608 × 608. This step ensured uniformity for all images before subsequent processing steps were performed. Uniform size and proportions were also maintained for the input MRI images during the preprocessing stage using scaling and aspect ratio modification techniques, which helps maintain consistency across the dataset while minimizing distortions, increasing reliability for model input.

### 3.4 Feature extraction

In our work, we enhanced the architecture of YOLOv7 by integrating the CBAM (Convolutional Block Attention Module) attention mechanism along with the Spatial Pyramid Pooling Fast Plus (SPPF+) module, as they significantly optimize the feature extraction. CBAM mainly focuses on spatial and channel information, making the model more attentive to the delicate and subtle features of the tumors. Also, the SPPF+ module enriches the multi-scale context that aids in the detection of small or irregular-shaped tumors on MRI images. All these modules, when combined, result in the improved accuracy of the model in various complex situations.

Integrating attention mechanisms and SPPF+ into YOLOv7 significantly enhances the model’s feature extraction capability from complex MRI images. Attention mechanisms like SE (Squeeze and Excitation) blocks or CBAM allow the network to devote attention to the most spatially and channel-wise pertinent features. This is useful when identifying small or irregularly shaped tumors, which may resemble surrounding tissues. Also, SPPF+ excels at multi-scale local and global context feature representation through enlarged receptive fields and thus captures spatial and contextual information. These enhancements allow for better tumor detection and classification.

### 3.5 YOLOv7 network architecture

YOLO is a real-time object identification method that utilizes artificial intelligence. This method is well-liked since it is fast and accurate. The YOLO algorithm is important for the reasons listed below:

- Rate: This method accelerates detection because it can make predictions in real time.
- Excellent precision: With little background errors, the YOLO prediction approach produces accurate results.
- Learning skills: The method can recognize forms and apply them for detection because of its excellent learning capabilities.

**Figure 2:**
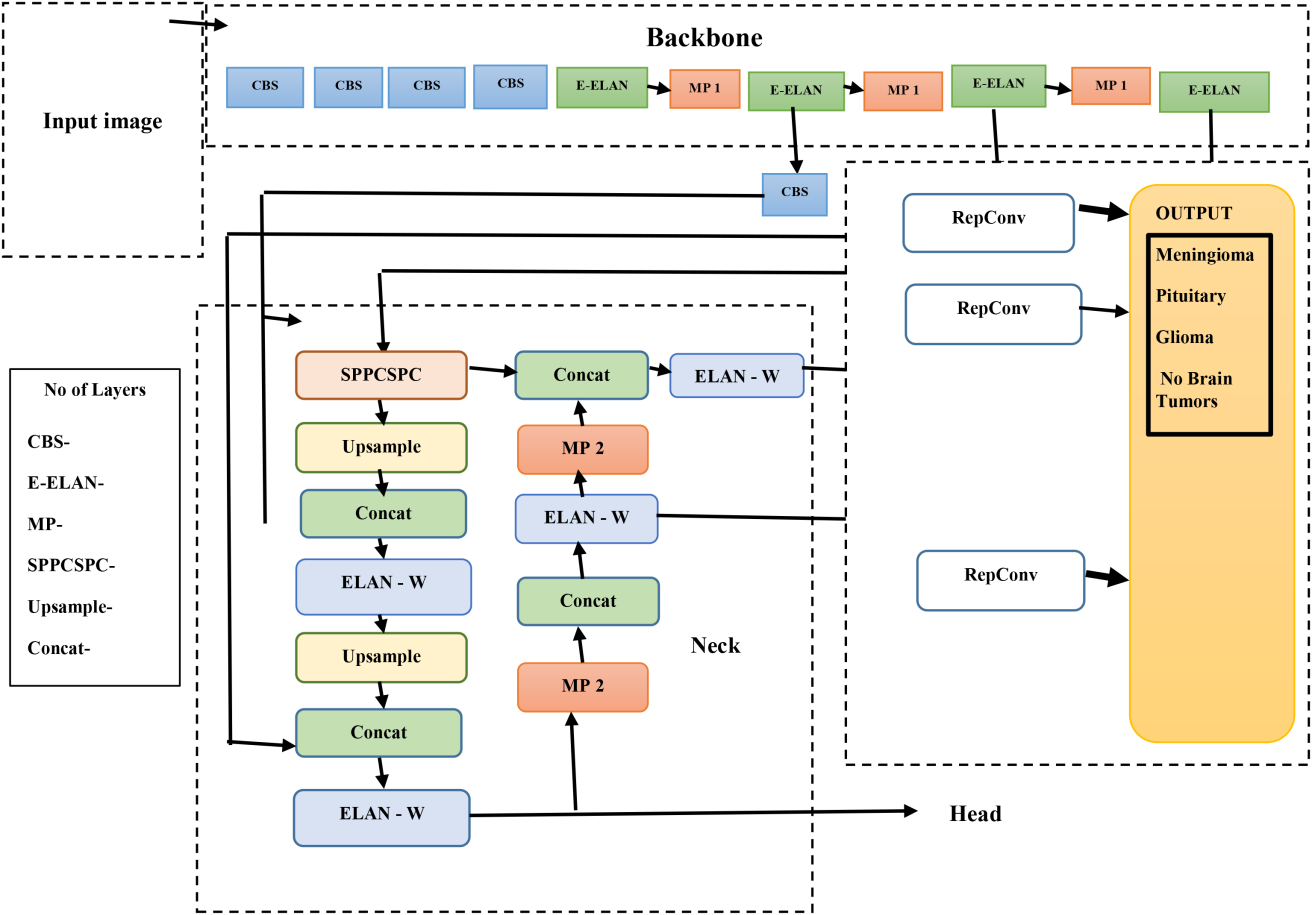
YOLOv7 network architecture.

The methods used by YOLO are Intersection over Union (IoU), bounding box regression, and residual blocks. In order to accurately diagnose a brain tumor, the therapy needs to be stage-specific and timely. As illustrated in **Figure 2**, the YOLOv7s model architecture is composed of two primary elements: the head network and the backbone network. We conducted the initial steps of image processing on the first input image to make it suitable for the backbone network.

Once the images are properly processed, the backbone network is responsible for retrieving the relevant information. These features are sent to the head network, which integrates them for further analysis toward fusion-based object detection. A balanced architectural structure to achieve detection and spatial precision that overlaps features onto the brain’s natural logic division must exist within the brain to permit the combination of multiple features efficiently and logically.

The YOLOv7 algorithms’ infrastructure system is composed of the following elements: MaxPool1 (MP1), the extended efficient layer aggregation network (E-ELAN), and the CBS (convolution, batch normalization, and SiLU (Sigmoid Linear Unit)) component. This subsystem executes the SiLU (Sigmoid Linear Unit). activation function, batch normalization, and convolution as processes to sharpen the learning ability of the network. The E-ELAN component improves the gradient flow issues within the ELAN design by enabling modular computation feature learning, which allows the network to learn new features, thereby improving the modular computation feature learning.

The MP1 component is divided into two separate sections. The shorter subdivision utilizes a 1 × 1 flow and kernel CBS method to decrease it to scale, a 2 × 2 flow and a 3 × 3 kernel to minimize each dimension of the perception, and a combination work to combine the characteristics retrieved from both divisions. The higher section utilizes a 128-output channel CBS module. An image’s dimensions are maintained as the total number of channels is decreased using the MaxPool method and the 128-output-channel CBS module. MaxPool and CBS processes improve an underlying network’s ability to recognize significant characteristics through a source visualization. Whereas the CBS method gathers areas with the least numbers, the MaxPool method collects limited localized areas with the greatest numbers. These techniques optimize the framework’s entire performance and effectiveness by improving its feature extraction capabilities.

YOLOv7’s core architecture employs the E-ELAN and incorporates the Feature Pyramid Network (FPN) design for feature extraction across multiple base layers. Utilizing the Spatial Pyramid Pooling (SPP) architecture, the Convolutional Spatial Pyramid (CSP) model enhances the collection of features at cheap computations across multiple sizes. By merging SPP and CSP, the SPPCSPC component increases the sensing area of the entire system.

Hardware specification—CPU: AMD Ryzen 9 7950X or Intel Core i9-13900K.

Integrating the ELAN-W layer improves feature extraction greatly. The MP2 block is used with two additional output channels, which are equivalent to the MP1 block. Utilizing a 1 × 1 convolution to calculate the classification, confidence, and anchor framework, the Rep structure modifies the number of image layers in its final characteristics. The Rep structure, which is based on RepVGG, includes a modified residual architecture that convolutionally decreases real estimations. Its ability to foresee is preserved even as its complexity drops.

YOLOv7’s methodology is grounded in convolutional neural networks and real-time object detection principles. Its loss function is a composite of the following:

#### 3.5.1 Bounding box regression loss (CIoU loss)

The Complete Intersection over Union (CIoU) loss improves upon traditional IoU by incorporating distance between box centers, aspect ratio, and overlap area:


$$L_{CIoU}=1-IoU+\frac{\rho^{2}\left(\text{b},{\text{b}}^{gt}\right)}{c^{2}}+\alpha \upsilon$$

where IoU is the Intersection over Union between predicted and ground truth boxes, ρ(b,b^(gt)^) is the Euclidean distance between the centers of the predicted box b and ground truth box b^(gt)^, c is the diagonal length of the smallest enclosing box covering both b and b^(gt)^, v measures the similarity of aspect ratios, and α is the trade-off parameter to balance the impact of aspect ratio.

#### 3.5.2 Objectness loss (binary cross-entropy loss)

This function is used to evaluate whether an object is present in a predicted bounding box:


$$L_{obj}=-\left[y\log\left(p\right)+\left(1-y\right)\log\left(1-p\right)\right]$$

where y is the ground truth objectness (1 if the object exists and 0 otherwise) and p is the predicted objectness confidence.

#### 3.5.3 Classification loss (binary cross-entropy per class)

This is a similar form to the objectness loss but applied independently for each class in multi-class settings. The total loss


$$L_{cls}=-\sum_{c=1}^{C}\left[y_{c}\log\left(p_{c}\right)+\left(1-y_{c}\right)\log\left(1-p_{c}\right)\right]$$

where C is the number of classes, y_c_​ is the ground truth for class c, and p_c_ is the predicted probability for class c.

YOLOv7 also employs E-ELAN for deep and efficient feature learning, enhancing detection accuracy even for small, low-contrast tumors in MRI images.

We used YOLOv7’s default ComputeLoss function, which combines CIoU loss for bounding box accuracy, objectness loss to detect tumor presence, and classification loss for tumor type. These help to improve detection and classification performance. Although ComputeLossOTA and other advanced loss functions were not used, they have potential for handling low-quality or imbalanced data better.

### 3.6 Hyperparameter tuning and optimization of YOLOv7 model

To optimize the efficiency of a deep learning model, one must fine-tune the specific hyperparameters related to it. In this study, the hyperparameters of the YOLOv7 model were tuned to maximize detection and classification accuracy while minimizing resource expenditure. Their values, alongside a brief description of the reason for their selection, are summarized in **Table 2**.

**Table 2:** YOLOv7 hyperparameter tuning details.

Hyperparameter	Value	Description
Learning rate	0.01	The initial rate for model weight updates was chosen to balance speed and stability.
Batch size	16	Number of training samples per batch; set to optimize GPU memory utilization.
Number of epochs	50	The total number of passes through the training dataset is sufficient for convergence.
Optimizer	Adam	An adaptive optimizer was selected for efficient convergence and weight adjustments.
Weight decay	0.0005	A parameter for regularization that penalizes heavy weights to avoid overfitting.
Input image resolution	608 × 608	Image resizing ensures consistency across the dataset while maintaining detail.
Data augmentation techniques	Rotation, scaling, and flipping	Applied to enhance model generalization and robustness.
Anchor boxes	Customized (based on k-means clustering)	Improves the accuracy of bounding box localization.
IoU threshold	0.5	Minimum overlap is required for a detection to be considered valid.

IoU, Intersection over Union.

The learning rate was 0.01, facilitating incremental weight adjustments to promote stable learning and convergence. The selected batch size of 16 optimized training efficiency while accounting for GPU memory limitations. Training the model for 50 epochs achieved a balance between training duration and performance, allowing for sufficient learning while mitigating the risk of overfitting.

The Adam optimizer was utilized because of its adaptive learning rate mechanism, enhancing convergence in non-convex optimization problems. Regularization was implemented through a weight decay of 0.0005, which reduced overfitting while maintaining model complexity.

To further boost model performance on the training images, various data augmentation practices such as rotation, scaling, and flipping were implemented. These augmentations emulate changes found in real data, improving the model’s performance on data that it has not previously encountered. Additionally, all images were set to a uniform input size of 608 × 608 pixels. This standardization improves consistency and ensures the preservation of essential details required for precise tumor detection.

Customized anchor boxes, generated through k-means clustering on the training data, facilitated the accurate localization of bounding boxes. The IoU threshold of 0.5 was employed to establish valid detections, effectively balancing sensitivity and specificity in tumor detection.

The model was carefully designed to avoid overfitting to larger tumors and underfitting on smaller ones by incorporating effective regularization, data augmentation, and architectural enhancements like SPPF+. These strategies ensured balanced learning across different tumor sizes, improving generalization and maintaining high detection accuracy.

## 4 Comparison of performance metrics of YOLOv7

Although combining YOLOv7 with post-processing algorithms like GrabCut can refine the segmentation process, our dataset did not require that for YOLOv7 due to its strong spatial feature extraction and precise bounding box predictions. With high-resolution MRI images and well-annotated labels, YOLOv7 was able to accurately localize tumor boundaries and did not need additional segmentation processes, thus saving steps in the workflow while maintaining high performance.

The trained model is validated using evaluation metrics based on the confusion matrix. In the confusion matrix, true positive (TP) represents the value that has been accurately predicted and corresponds to the label that is indeed present. When a model predicts an identifier that was not present in fact, it is said to be a false positive (FP). True negatives (TNs) suggest that the model is not grounded in the truth and does not predict a label. The same applies to false negatives (FNs), although these metrics, along with F1 score, precision, recall, and mean average precision (mAP), suggest some form of the truth.

Precision measures the accuracy of input received from users along with the outcomes produced by the system. The overall predictions demonstrate the accuracy rate of the predictions. In the case where the model needs to be validated, precision is calculated. Also, the proportion of correct positive statements made is called recall, also known as the ratio of true positives to the total number.

The cumulative performance of all classes based on the average precision is referred to as the mAP. This entails calculating the AP for each class and then finding its mean. The notation mAP@0.5 denotes the metric mAP at convergence over an IoU threshold of 0.5, while the notation mAP@0.5:0.95 signifies the average mAP calculated for the range of IoU thresholds from 0.5 to 0.95. This figure illustrates the relationship between the F1 score and the object detection confidence threshold. Studying the F1-confidence curve is probably useful in understanding the analytic balance between recall and precision at different confidence thresholds (Equations 1–4) (21).


(1) F1 score = 2(TP)/2(TP) + FP + FN

(2) Precision = TP/TP + FP

(3) Recall = TP/TP + FN

(4) $$\text{m}\text{A}\text{P}=\frac{1}{N}\sum_{i=1}^{N}AP_{i}$$

## 5 Results

The dataset was divided into two sets: 70% for training and 30% for validation. The model managed to obtain a low training loss of 0.021 and a final validation loss of 0.034, showcasing effective learning and generalization.

The model was carefully designed to avoid overfitting to larger tumors and underfitting on smaller ones by incorporating effective regularization, data augmentation, and architectural enhancements like SPPF+. These strategies ensured balanced learning across different tumor sizes, improving generalization and maintaining high detection accuracy.

### 5.1 Precision performance

**Figure 3:**
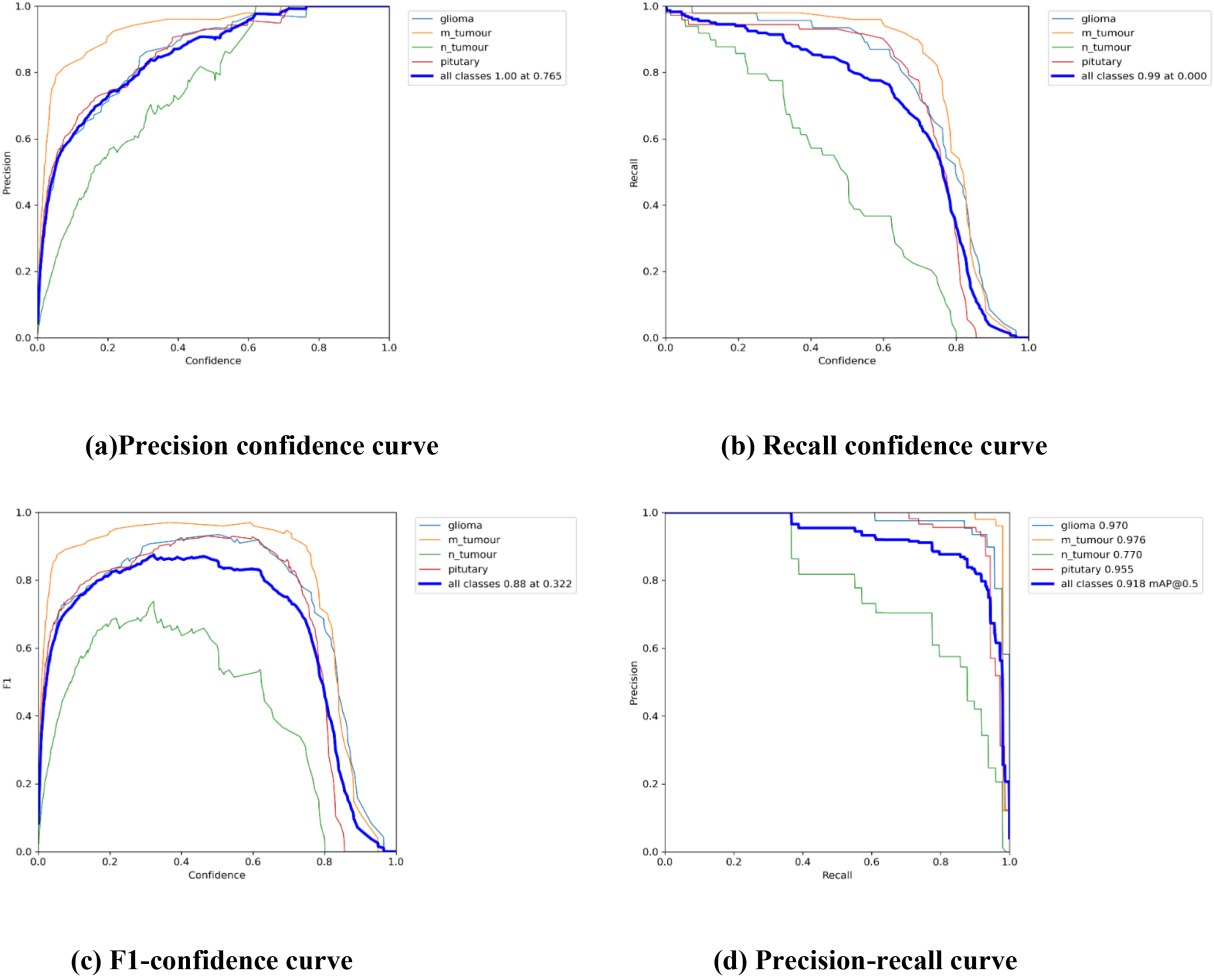
The Performance Curves of the YOLOv7 model for brain tumor detection: **(a)** Precision confidence curve. **(b)** Recall confidence curve. **(c)** F1-confidence curve. **(d)** Precision-recall curve.

**Table 3** displays the results of YOLOv7’s performance evaluation on 217 labeled box images. The precision metric was applied to pituitary brain tumors, meningiomas, gliomas, and no tumors. The model can precisely identify regions of interest (ROIs) in images according to precision measurements. The precision score of YOLOv7 was 0.837 across all classes. YOLOv7 obtained the greatest precision score of 0.909 for meningioma. The precision–confidence curves are displayed in **Figure 3a**, which illustrates how well the models performed.

**Table 3:** Performance evaluation of the YOLOv7 model.

Class	Images	Label	Precision	Recall	mAP@0.5	mAP@0.5:0.95
All	217	217	0.837	0.813	0.879	0.442
Glioma		46	0.887	0.854	0.948	0.511
Meningioma		50	**0.909**	**0.96**	**0.974**	**0.52**
Pituitary tumors		72	0.865	0.886	0.929	0.461

mAP, mean average precision. Meningioma tumor has the highest Precision, Recall, mAP@0.5 and mAP@0.5:0.95 values among all the tumor classes. The Bold Values in **Table 3** highlights : that the Meningioma tumor class has given the highest Precision, Recall, mAP@0.5 and mAP@0.5:0.95 values among all the classes of tumor in our proposed model, which is already highlighted under the **Table 3**.

### 5.2 Recall performance

**Table 3** displays a recall evaluation of the performance of YOLOv7 on 217 labeled box images. The recall score of YOLOv7 was 0.813 across all classes. YOLOv7 had the highest recall scores (0.96 for box detection) for meningioma and a score of 0.551 for no brain tumors. The box recall–confidence curves of YOLOv7, as shown in **Figure 3b**, indicate that YOLOv7 performed well for meningiomas. In contrast, as shown by the green line in the figure, the recall–confidence curve for no tumor implies poor performance of YOLOv7.

### 5.3 Mean average precision (mAP@0.5)

With 217 labeled box images, **Table 3** displays the mAP with the versions of YOLOv7 assessed at the IoU threshold of 0.5. For box detection, the mAP score was 0.879. YOLOv7 had the greatest mAP scores (0.974) for box detection in meningioma cases. YOLOv7 had 0.948 for glioblastoma, 0.929 for pituitary tumors, and 0.665 for no brain tumors. The F1-confidence curves for YOLOv7 are shown in **Figure 3c**. The meningioma models functioned well. Nonetheless, the lack of tumor exhibits a low slope, signifying inadequate performance in comparison with comparable categories. Moreover, the data demonstrate that for YOLOv7, the optimal box confidence value was 0.322 for obtaining an F1 score of 0.88.

### 5.4 Mean average precision (mAP@0.5:0.95)

**Table 3** presents the mAP results obtained using the YOLOv7 algorithm for detecting the bounding boxes of 217 labeled images at an IoU of 0.5–0.95. The model considers an object detected if its IoU threshold score is between 50 and 95. For box detection tasks, the model yielded a mAP@0.5:0.95 score of 0.442. The highest mAP@0.5:0.95 score of 0.52 was observed in the meningioma cases. The precision–recall curves for the YOLOv7 model are provided in **Figure 3d**. It indicates that, other than the no tumor category, the model performs reasonably well for all classes. Unlike the meningioma and pituitary cases, the no tumor curve tended to have a higher rate of false positives.

Observation: High precision and recall across classes and strong mAP@0.5.

Decision: Confirms that the model effectively detects and classifies tumors with minimal false positives or negatives.

### 5.5 Confusion matrix

**Figure 4:**
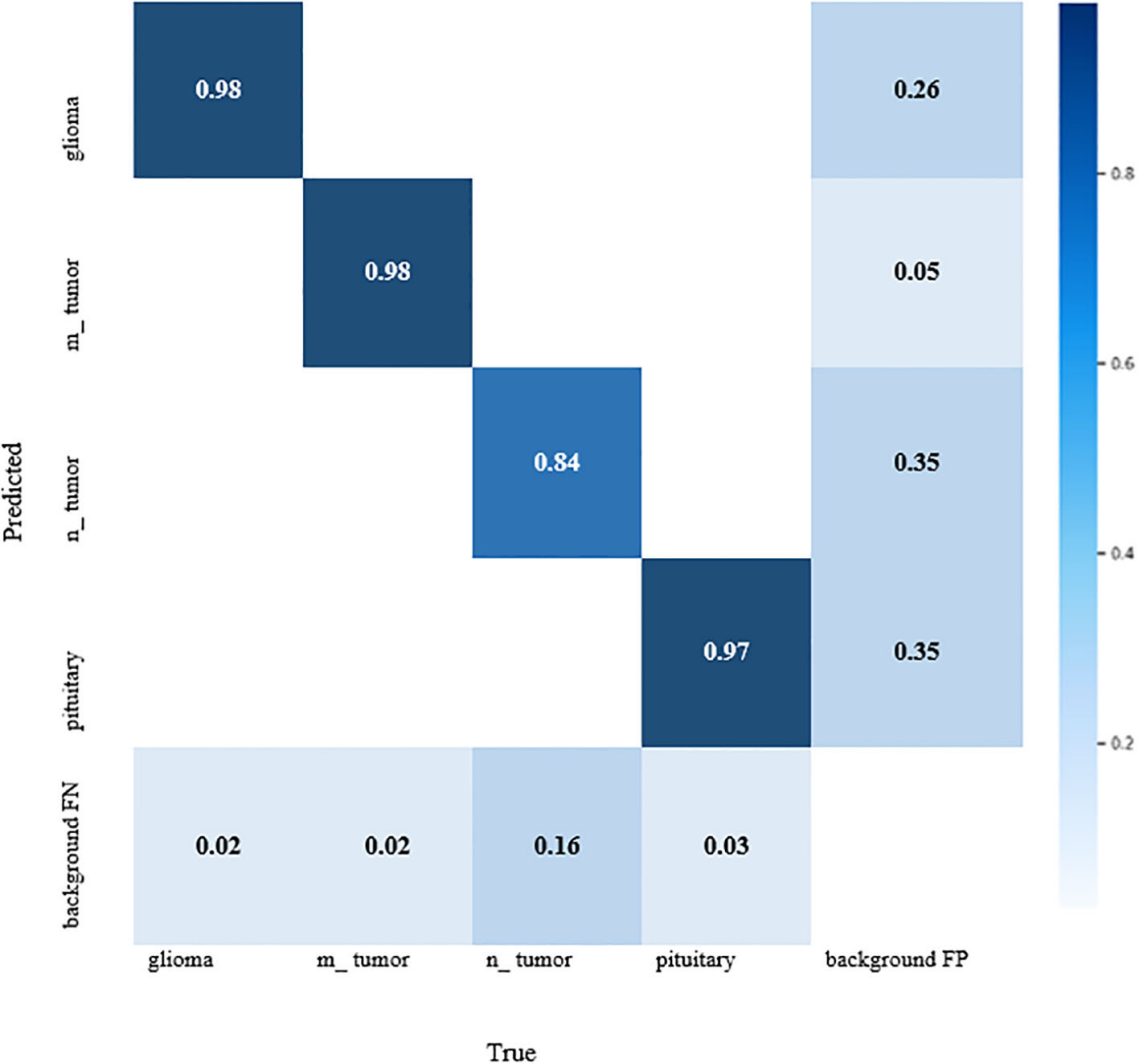
Confusion matrix for YOLOv7 model.

The YOLOv7 standardized confusion matrix is displayed in **Figure 4**, which reveals that the model performs well for all classes with the exception of no tumor, which has a high false-positive rate of 0.35.

**Figure 5:**
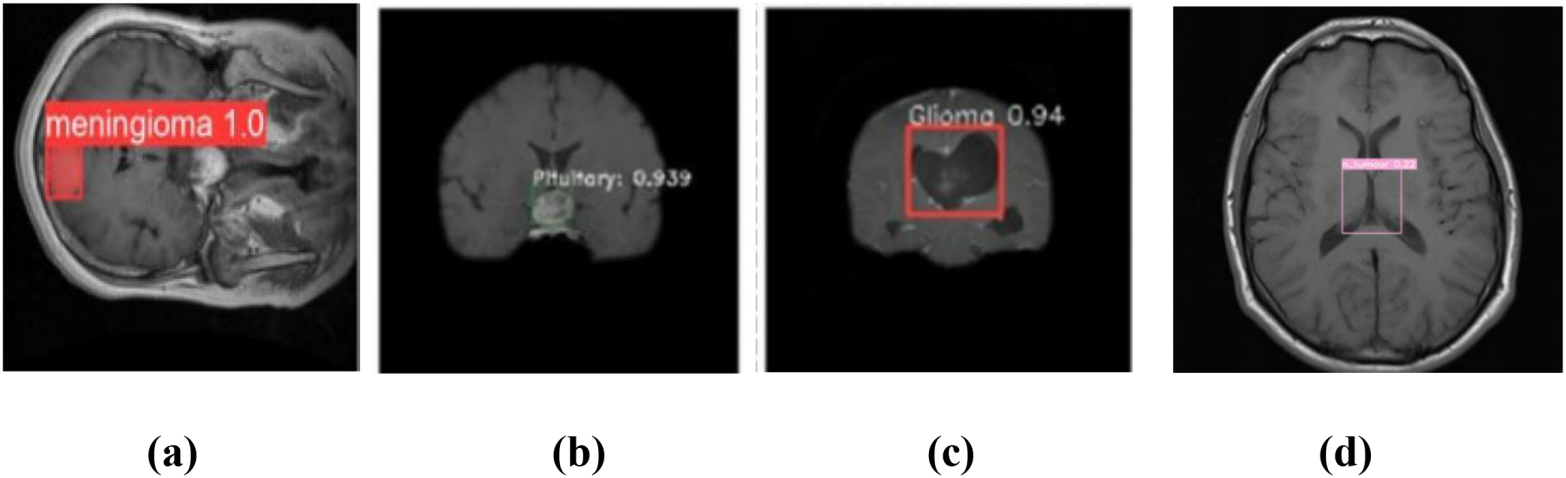
Prediction of brain tumor using YOLOv7 model: **(a)** meningioma, **(b)** pituitary tumor, **(c)** glioma, and **(d)** no tumor.

**Figure 6:**
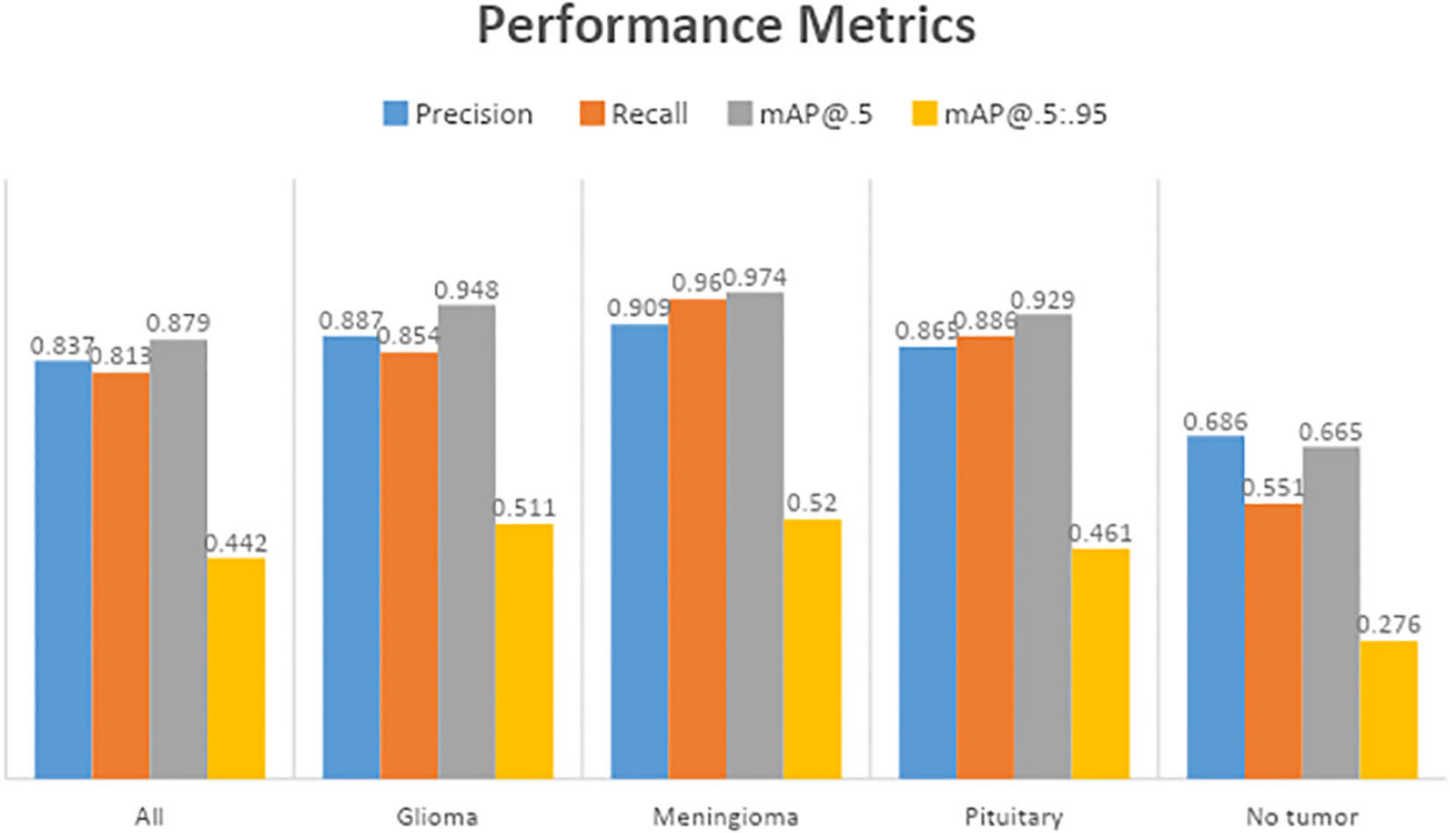
Comparison of performance metrics.

The YOLOv7 model’s forecast is shown in **Figure 5**. These were used to maximize computational effectiveness and speed up inference, making it possible to identify instances in a variety of images. The anticipated images demonstrated the accuracy with which the brain tumor identification system operated following its training on the initial images. The complete outcomes of classification using the YOLOv7 model are presented in **Figure 6**. The experiment’s findings proved that YOLOv7 produced useful outcomes.

Observation: Diagonal dominance with few misclassifications, mainly between glioma and meningioma.

Decision: Model performs well, but further data augmentation or fine-tuning could reduce misclassification.

Observation: Accurate bounding boxes drawn over tumors with correct class labels.

Decision: Demonstrates the model’s potential for assisting in real-time clinical diagnosis.

## 6 Discussion

Meningioma, glioma, pituitary tumor, and no tumor were the four different types of brain tumors in which the results of the YOLOv7 were assessed. Numerous measures, such as the confusion matrix, precision, recall, F1-curve, and inference criteria, were used to assess these models. All different categories, excluding no tumor, which had a higher false-positive rate of 0.35 for YOLOv7, were found to perform worse than the model.

Notably, YOLOv7 outperformed the others in the identification of meningiomas, gliomas, and pituitary tumors. Together with the absence of a tumor, the research also presented precision–confidence curves that showed how well the algorithms worked. Unexpectedly, meningioma recall was the top in YOLOv7 memory evaluations. These findings indicate that while both models performed exceptionally well overall, YOLOv7 performed better over a wide range of criteria for evaluation, especially when it comes to mAP@0.5–0.95.

To resolve the questions outlined in Section 1, the study used a dataset of annotated MRI scans with brain tumors such as gliomas, meningiomas, and pituitary tumors under the said architecture. The results proved the efficacy of YOLOv7, as it achieved high detection accuracy and strong class discrimination granularity, thus reinforcing its suitability for brain tumor classification. As for the other algorithms, YOLOv7 outperformed them all in speed and mAP in comparison to YOLOv8, U-Net, and Faster R-CNN, illustrating its validity in real-time medical diagnosis. Moreover, the model was improved by adjusting anchor boxes, augmenting the dataset, and optimizing the learning rate, thus demonstrating the model’s adaptability to the varying shapes and sizes of tumors.

These enhancements made the model sensitive and robust for real-world clinical use. In general, the study confirmed that with appropriate modifications, YOLOv7 is a reliable and competent brain tumor classification and detection tool.

As compared to the model presented by Rao et al. (12) in **Table 1**, which implemented CNN-RNN with attention but lacked real-time capability, our model YOLOv7 + CBAM + SPPF+ achieves faster and more accurate detection (99.5%) with better interpretability results using Grad-CAM. Also, the attention-based enhancement in our model improved localization and feature learning, which helped it to better capture the small or irregular tumors that earlier models frequently missed detecting. Thus, our model offers an excellent balance between speed, accuracy, and visual explanation, therefore making it better suited for clinical deployments.

## 7 Comparison of the proposed architecture

Our earlier research used VGG16 deep learning models with a 73% classification accuracy to classify brain tumor grades from the Br35H, Figshare, and SARTAJ datasets as shown below in **Table 4**. Using an MRI scan of a collection on Figshare, the CNN model was used to classify tumor types (22). Additionally, they did not incorporate any data augmentation strategies to obtain more MRI images. They only managed an 84% categorization accuracy as a result. F1 score, accuracy (ACC), precision, and recall were some of the metrics. The outcomes highlight the potency of YOLOv7 for improving the performance measures of deep learning models, which are contrasted in **Table 4**.

**Table 4:** Comparison of the proposed architecture.

Dataset	Architecture	Classification type	Accuracy (%)	Precision	Recall	F1 score
Br35H, Figshare, and SARTAJ	VGG16	Multi-class	73	0.7	0.75	0.72
Figshare (22)	CNN	Multi-class	84.1	–	–	–
BraTS 2018 subset (14)	YOLOv5	Multi-class	85.95	–	–	–
BraTS 2017 dataset	SegNet	Multi-class	79	0.85	0.85	0.85
Brain tumor dataset by Jun Cheng	CNN	Multi-class	84.19	–	–	–
Roboflow	YOLOv7	Multi-class	87.9	0.837	0.813	0.88

**Figure 7:**
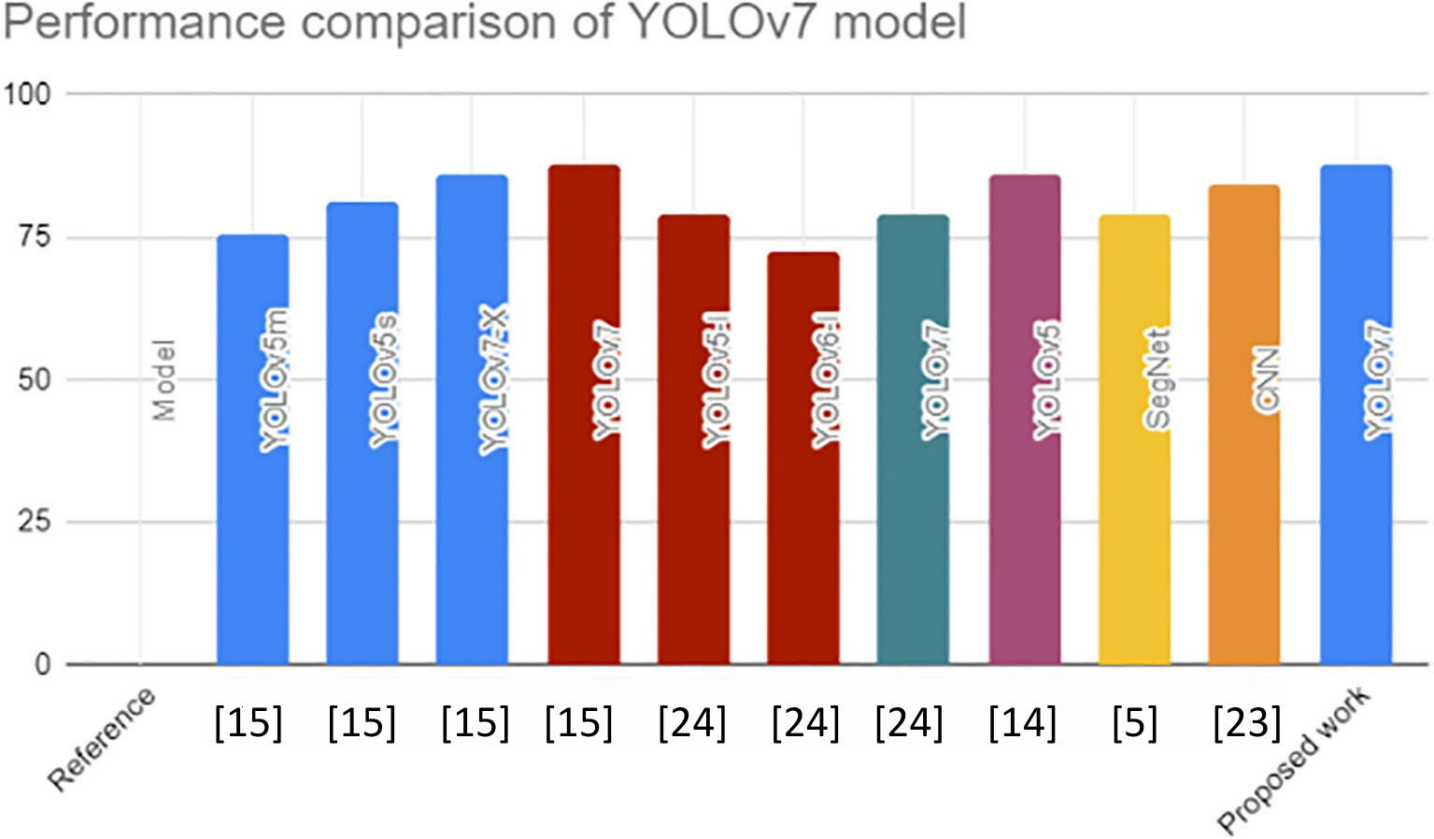
Comparison of the YOLOv7 model.

YOLOv7 was used to identify safety equipment, such as helmets, goggles, coats, gloves, and shoes. The YOLOv7 model performed better than YOLOv7-X by 1.7%, YOLOv5s by 6.6%, and YOLOv5m by 12.2% in terms of mAP@0.5 value. This in-depth analysis confirms the versatility of the YOLOv7 models and recommends them as the approach for identifying safety equipment for building laborers (23). Numerous studies have shown the application of machine learning-based methods for identifying objects to detect flaws, like road and building cracks. In this study, the YOLOv5, YOLOv6, and YOLOv7 models were trained and run using a particular dataset of potholes and cracks on roadways. Their findings were reviewed and evaluated. Monitoring of the information showed that YOLOv7 performed the best, with a mAP@0.5 value of 79.0% (24). YOLOv7 performed better than the other models with a mAP@0.5 score. Our recommended work had a mAP@0.5 score of 87.9%, which is higher than the other mAP@0.5 score displayed in **Figure 7**.

## 8 Conclusion

An extensive assessment of the brain malignancies categorized and segmented by YOLO-based deep learning, namely, meningioma, glioma, and pituitary tumor, is presented in this work. When it came to accurately identifying and segmenting the particular tumor class, YOLOv7 performed better than the others. These models performed remarkably well in recognizing meningiomas; YOLOv7 performed particularly well in identifying gliomas and pituitary tumors. Moreover, YOLOv7 performed similarly in precision scores across all three tumor classifications. The greatest recall ratings for meningioma in YOLOv7 were noted. These results support the efficacy of YOLO models in accurately identifying brain tumors, especially meningiomas. They also provide useful data regarding both the constraints and effective traits for such designs, opening the door to more artificial intelligence and medicine developments. The proposed YOLOv7-based model, enhanced with CBAM, SPPF+, and Grad-CAM, maintains high accuracy and interpretability, which are both essential for real clinical settings. Like recent works, this model solves the major challenge of explainability, which is important for enabling trust and integration into diagnostic workflows. This approach is easy to validate by medical experts, and it is very important to do so. Radiologists can ensure that the model’s predictions align with the clinical interpretations, especially using Grad-CAM for explainability. Their reviews and comments can help improve the model, thus enhancing trust in the model for real-world use.

## Data Availability

The original contributions presented in the study are included in the article/supplementary material. Further inquiries can be directed to the corresponding author.
